# Follow-Up of Adefovir Dipivoxil Induced Osteomalacia: Clinical Characteristics and Genetic Predictors

**DOI:** 10.3389/fphar.2021.636352

**Published:** 2021-04-28

**Authors:** Jiao Zhao, Wei-guang Feng, Zhe Wei, Jian Zhou, Xiao-yun Chen, Zhen-lin Zhang

**Affiliations:** ^1^ Shanghai Clinical Research Center of Bone Disease, Department of Osteoporosis and Bone Disease, Shanghai Jiaotong University Affiliated Sixth People’s Hospital, Shanghai, China; ^2^ Department of Hepatology, The Fourth People's Hospital of Huai'an, Huai'an, China; ^3^ Department of Rheumatology, Shanghai University of Traditional Chinese Medicine Affiliated LongHua Hospital, Shanghai, China

**Keywords:** adefovir dipivoxil, drug-related side effects, osteomalacia, pharmacogenomic variants, clinical characteristic

## Abstract

Adefovir dipivoxil (ADV) is widely used for chronic hepatitis B therapy in China. To explore the clinical features and prognosis of ADV-induced osteomalacia and to analyze the association between osteomalacia and genetic variants in 51 drug transporters genes. Clinical and follow-up data of the ADV-treated patients were collected. Target capture sequencing was used to identify genetic variations of 51 drug transporter genes. A total of 193 hepatitis B patients treated with ADV were enrolled, of whom 140 had osteomalacia. The other 53 without osteomalacia were included in the control group. The median duration of ADV treatment before the onset of osteomalacia was 6.5 years (range:1.5–7 years). We found that most patients with osteomalacia had hypophosphatemia, high serum alkaline phosphatase levels, hypouricemia, nondiabetic glycosuria, proteinuria. Stopping ADV administration, supplementing calcitriol and calcium were effective treatments. During 3–6 months of follow-up, the clinical symptoms and biochemical indicators of patients with osteomalacia have been significantly improved. There was no significant difference in duration of adefovir treatment in patients with or without osteomalacia (*p* = 0.791). Through regression analysis, we found that age was a risk factor for osteomalacia [per 1 year, odds ratio (OR), 1.053; 95% confidence interval (95% CI), 1.020–1.087; *p* = 0.015]. 1992 single nucleotide variants were found using target capture sequencing. However, the associations of genetic variants of 51 drug transporter genes and the risk of osteomalacia were negligible. Osteomalacia is prone to occur in patients with chronic hepatitis B treated with long-term ADV at a therapeutic dose. After standard treatment, the prognosis is mostly good. We failed to find genetic variants that can predict the risk of ADV-induced osteomalacia.

## Introduction

Adefovir dipivoxil (ADV), a type of nucleotide analogue developed in 2002, is widely used for chronic hepatitis B therapy (Hadziyannis et al., 2003; Marcellin et al., 2003; Han, 2006; Chen an Ju, 2012). Several side effects associated with ADV are reported, including acute renal failure and Fanconi syndrome (Izzedine et al., 2009; Jung et al., 2010; Girgis et al., 2011). These side effects have been well known to occur at a dose of 60 mg/d or 120 mg/d when treating human immunodeficiency virus (Earle et al., 2004; Izzedine et al., 2004; Hadziyannis et al., 2006; Izzedine et al., 2009). The first case of acquired Fanconi syndrome associated with a therapeutic dose of ADV (10 mg/d) was published in 2008 (Lee et al., 2008). In recent years, several cases have been reported showing that long-term application of a low dose of ADV causes kidney tubular dysfunction (KTD) and osteomalacia (Wong et al., 2010; Kim et al., 2013; Law et al., 2013; Shimohata et al., 2013; Wu et al., 2013; Jeong et al., 2014; Wang et al., 2015). In China, ADV is still a frequently used antiviral drug, especially in the underdeveloped midwestern area. Thus, the State Food and Drug Supervision and Administration has warned that attention should be paid to the risk of ADV-induced osteomalacia (Zhu et al., 2018).

The mechanism by which ADV causes the KTD and osteomalacia is not fully understood. Abnormality of drug transporters can lead to the accumulation of ADV in the renal tubular epithelial cells. As a result, renal tubular epithelial cells are damaged, leading to KTD and osteomalacia. The function of drug transporters is an important factor for kidney damage caused by ADV (Wei et al., 2016). Moreover, many studies have revealed that osteomalacia induced by long-term application of ADV has significant individual differences (Kahn et al., 1999), and ADV-induced osteomalacia is mostly reported in the Asian population (Kim et al., 2012; Tanaka et al., 2014; Wei et al., 2016). These evidences indicate that genetic factors may play a very important role in the development of tubular dysfunction induced by ADV. Therefore, it is necessary to conduct pharmacogenetic research on the occurrence of ADV-related adverse reactions.

Although ADV induced osteomalacia has been reported in recent years, there is a lack of long-term follow-up data. The prognosis of this disease remains to be clarified. In addition, there have been few studies on the genetic risk factors of ADV induced KTD and osteomalacia. We have studied the clinical features and genetic predictors of 76 patients with ADV-induced hypophosphatemic osteomalacia in 2016 and found a higher percentage of the GA genotype at rs3740070 of the *ABCC2* gene in patients than in healthy people and three missense mutations predicted to have a pathogenic effect (Wei et al., 2016), but we lacked strict control groups. As a result, in this study we expanded the sample size and introduced a control group. 193 ADV-treated hepatitis B patients were enrolled, of which 53 were in the control group. Through an average follow-up of 11.7 months, we analyzed the clinical features and prognosis of this disease, and performed target capture sequencing of 51 transporter genes to explore the association between ADV-associated osteomalacia and variants in genes encoding drug transporters.

## Materials and Methods

### Study Subjects

From June 2008 through May 2018, 193 patients were treated in the Shanghai Jiao Tong University Affiliated Sixth People’s Hospital and Huaian First People’s Hospital. All study subjects were chronically infected with hepatitis B virus and were treated with low-dose ADV (10 mg/d). All study subjects have excluded people with diseases known to affect bone metabolism or renal function, and those who have taken various drugs that affect bone metabolism. Inclusion criteria of the case group were as follows: 1) presence of core criterion, including symptoms of fatigue and bone pain, hypophosphatemia or high serum alkaline phosphatase (ALP); 2) presence of at least two supplemental criteria, including X-ray suggesting fracture and pseudo-fracture, nondiabetic glycosuria and/or proteinuria, hypouricemia, hypokalaemia, compensated or decompensated metabolic acidosis. Patients in the control group had no related symptoms and abnormal biochemical examinations. A total of 140 patients were diagnosed with ADV-induced osteomalacia and included in the case group. At the same time, another 53 patients were included in the control group. In the case group, 30.9% (30 of 97 patients) were treated with imported ADV, 61.9% (60 of 97 patients) with domestically produced ADV, and 7.2% (7 of 97 patients) used alternately. As for the control group, 26.4% (14 of 53 patients) took imported ADV, and the remaining patients took domestic ADV. There was no difference in the brand of the drug between the two groups (*p* = 0.085).

### Data Collection

After obtaining a detailed medical and family history (including history of hepatitis B and adefovir treatment), the following were tested to make a diagnosis: arterial blood gas, urine routine markers, hepatic and renal function, serum electrolytes. X-ray radiography was performed if necessary. A Lunar Prodigy dual-energy X-ray absorptiometry densitometer (Lunar Corporation, Madison, WI, United States) was used to obtain the bone mineral density (BMD) values of the left proximal femur, including the femoral neck, total hip, and anteroposterior lumbar spine 1–4 (L1–4). Whole-body bone images were obtained by single-photon emission computed tomography (SPECT)/CT on 39 patients. Patients were followed for an average of 11.7 months (1–111 months) after therapy. During follow-up, serum phosphorus, serum ALP, urine routine markers, beta C-terminal cross-linked telopeptides of type l collagen (β-CTX) and serum osteocalcin in the form of N-terminal midmolecule fragment (OC) were tested.

### Candidate Genes Selection

A variety of drug transporters have been identified, and these transporters are mainly distributed on the renal proximal tubular epithelial cells, which are involved in the intake and excretion of drugs (Masereeuw and Russel, 2004; König et al., 2013; Wong et al., 2018). Proteins reported to be involved in the transport of ADV include organic anion transporter family (OAT), multidrug resistance-associated protein (MRP) and multidrug resistance (P-glycoprotein) family (MDR). OAT is responsible for transporting ADV into tubular epithelial cells (ingestion), while the latter two transport ADV out of the cells (excretion). The above transporters and their coding genes are related to the metabolism of ADV (Fujita et al., 2005; Izzedine et al., 2005; Mallants et al., 2005; Imaoka et al., 2007; Uwai et al., 2007; Nieskens et al., 2016). In this study, we selected 51 drug transporter encoding genes on the basis of their functional significance and findings of previously published studies (Fotiadis et al., 2013; Wright, 2013; Young et al., 2013; Markovich, 2014; Nigam et al., 2015; Wall and Lazo-Fernandez, 2015; Chen et al., 2016; Klein and Sands, 2016; Ivanyuk et al., 2017; Lefevre and Boutry, 2018; Xu et al., 2018; Levi et al., 2019; Yang and Han, 2019). The transporters encoded by these genes play an important role in drug transport and are expressed on the apical or basolateral of renal tubular epithelial cells. The basic characteristics of these 51 genes are shown in Table 1.

**TABLE 1:** Drug transporters and their coding genes.

Gene	Protein	Tissue distribution (cellular/subcellular expression)	Gene locus
*ABCB1*	MDR1	Adrenal, kidney, brain (apical in epithelial cells)	7p21
*ABCB4*	MDR3	Liver, kidney, adrenal gland, heart (apical in epithelial cells)	7q21.1
*ABCB11*	BSEP	Liver, kidney (apical in epithelial cells)	2q24
*ABCC1*	MRP1	Lung, testis, peripheral blood mononuclear cells, kidney (basolateral in epithelial cells)	16p13.1
*ABCC2*	MRP2	Liver, kidney, intestine (apical in epithelial cells)	10q24
*ABCC3*	MRP3	Liver, intestine, colon, prostate, testis, brain, kidney (basolateral in epithelial cells)	17q21.3
*ABCC4*	MRP4	Ubiquitous (apical in epithelial cells)	13q32
*ABCC5*	MRP5	Ubiquitous (basolateral in epithelial cells)	3q27
*ABCC6*	MRP6	Kidney, liver (apical in epithelial cells)	16p13.1
*ABCC10*	MRP7	Ubiquitous (apical and basolateral in epithelial cells)	6p21
*ABCG2*	BCRP	Placenta, intestine, liver, colon, kidney (apical in epithelial cells)	4q22
*RHCG *	RhCG	Kidney, brain, testis, placenta, pancreas, prostate (apical in epithelial cells)	15q26.1
*SLC2A9 *	GLUT9	Kidney, liver, placenta, lung (basolateral in epithelial cells)	4p16.1
*SLC4A8 *	NDCBE	Brain, spinal column, testis, trachea, thyroid, kidney (basolateral in epithelial cells)	12q13.13
*SLC5A2 *	SGLT2	Kidney, brain, liver, heart muscle, thyroid, salivary glands (apical in epithelial cells)	16p11.2
*SLC5A8 *	SMCT1	Intestine, kidney, brain, retina, muscle (apical in epithelial cells)	12q23.1
*SLC5A12 *	SMCT2	Intestine, brain, retina, muscle, kidney (apical in epithelial cells)	11p14.2
*SLC6A18 *	B (0) AT3	Kidney (apical in epithelial cells)	5p15.33
*SLC7A8 *	LAT2	Intestine, kidney, lung, heart, spleen, liver, brain (basolateral in epithelial cells)	14q11.2
*SLC7A9 *	b (0,+) AT	Kidney, intestine, liver, placenta (apical in epithelial cells)	19q13.1
*SLC9A3 *	NHE3	Kidney (apical in epithelial cells)	5p15.33
*SLC13A1 *	NAS1	Kidney, intestine (apical in epithelial cells)	7q31.32
*SLC14A2*	UT2	Kidney (apical and basolateral in epithelial cells)	18q12.3
*SLC15A1*	PEPT1	Liver, kidney, intestine (apical in epithelial cells)	13q32.2-q32.3
*SLC15A2*	PEPT2	Kidney, intestine (apical in epithelial cells)	3q13.33
*SLC22A1*	OCT1	Liver, intestine, lung, heart, placenta, kidney (basolateral in epithelial cells)	6q25.3
*SLC22A2*	OCT2	Liver, kidney, brain, intestine (basolateral in epithelial cells)	6q25.3
*SLC22A3*	OCT3	Liver, intestine, pancreas, brain, heart, kidney (basolateral in epithelial cells)	6q25.3
*SLC22A4*	OCTN1	Kidney, liver, testis (apical in epithelial cells)	5q31.1
*SLC22A5*	OCTN2	Kidney, liver, brain, intestine, testis (apical in epithelial cells)	5q31.1
*SLC22A6*	OAT1	Kidney, brain, eye (basolateral in epithelial cells)	11q12.3
*SLC22A7*	OAT2	Liver, kidney (basolateral in epithelial cells)	6p21.1
*SLC22A8*	OAT3	Blood cerebrospinal fluid barrier, liver, kidney, eye (basolateral in epithelial cells)	11q12.3
*SCL22A11*	OAT4	Liver, placenta, kidney (apical in epithelial cells)	11q13.1
*SLC26A1 *	SAT1	Liver, kidney, intestine (basolateral in epithelial cells)	4p16.3
*SLC26A4 *	Pendrin	Thyroid, kidney, brain (apical in epithelial cells)	7q22.3
*SLC26A6 *	CFEX	Pancreas, kidney, heart (apical in epithelial cells)	3p21.31
*SLC26A7 *	SUT2	Stomach, kidney (apical in epithelial cells)	8q21.3
*SLC28A1 *	CNT1	Kidney, liver, intestine (apical in epithelial cells)	15q25.3
*SLC28A2 *	CNT2	Heart, muscle, liver, kidney, intestine, pancreas (apical in epithelial cells)	15q15
*SLC28A3 *	CNT3	Ubiquitous (apical in epithelial cells)	9q22.2
*SLC29A1 *	ENT1	Ubiquitous (apical in epithelial cells)	6p21.1
*SLC29A2 *	ENT2	Ubiquitous (basolateral in epithelial cells)	11q13
*SLC34A1 *	NPT2a	Kidney, bone (apical in epithelial cells)	5q35.3
*SLC47A1 *	MATE1	Adrenal gland, liver, muscle, kidney (apical in epithelial cells)	17p11.2
*SLC47A2 *	MATE2	Kidney, brain (apical in epithelial cells)	17p11.2
*SLC O 1A2*	OATP1A2	Brain, kidney, liver, intestine (apical in epithelial cells)	12p12
*SLC O 2B1 *	OATP2B1	Liver, placenta, intestine, heart, skin, kidney (basolateral in epithelial cells)	11q13
*SLC O 3A1*	OATP3A1	Ubiquitous (basolateral in epithelial cells)	15q26
*SLC O 4A1*	OATP4A1	Ubiquitous (basolateral in epithelial cells)	20q13.1
*SLC O 4C1 *	OATP4C1	Kidney (basolateral in epithelial cells)	5q21

### Pharmacogenetic Analyses

Since 35 patients refused to provide DNA, we finally obtained the DNA of 106 patients in the case group and 52 patients in the control group. DNA was isolated from peripheral blood leukocytes using the QuickGene DNA Whole Blood Kit L (KURABO, Japan) and the Nucleic Acid Isolation System (QuickGene-610L, FUJIFILM, Japan). We then used the target capture sequencing method to detect single nucleotide variant (SNV) in 51 drug transporter encoding genes. Gene detection range requirements was the exon region of the genes (5′ and 3′ untranslated sequences and coding region). After amplification of the purified and polymerase chain reaction, the DNA library was constructed. Then, the library was hybridized with the probe to capture the target region using an Agilent SureSelect XT custom kit (Agilent Technologies, CA, United States). Dynabeads® MyOne^TM^ Streptavidin T1 magnetic beads (Thermo Fisher Scientific, MA, United States) were used to enrich the DNA belonging to the region of interest. The original library was amplified using a high-fidelity polymerase and purified by magnetic beads so it would be suitable for sequencing. After quality assessment, the library was finally subjected to high-throughput sequencing in 2 × 150 bp double-end sequencing mode on the Illumina HiSeq platform (Illumina, CA, United States).

### Statistical Analyses

Descriptive results of continuous variables are expressed as medians and interquartile ranges. Parametric (Student’s t test) and non-parametric tests (Wilcoxon test) were used to compare continuous variables. Statistical comparisons for genotype frequencies between two groups were made by use of the χ^2^ test, and Fisher’s corrections were applied as required. Associations between genotypes and osteomalacia were tested by logistic regression analyses. We used odds ratios (ORs) and 95% confidence intervals (95% CIs) to estimate the impact of each variable on osteomalacia. To control the false discovery rate (FDR), the Benjamini-Hochberg method was used to adjust for multiple testing. Haploview software was used to test Hardy-Weinberg equilibrium. All other statistical analyses were performed using SPSS, version 22.0 (SPSS). Differences with *p* values < 0.05 were considered significant.

### Ethics Statement

The study protocol was approved by the Committee of the Ethics of Human Research in the Shanghai Jiaotong University Affiliated Sixth People’s Hospital. All patients provided informed consent for inclusion in the study according to the guidelines at our institution.

## Results

### General Characteristics

We obtained demographic and clinical information about all 193 patients. Some of the main characteristics are presented in Table 2. There was no significant difference in sex ratio (male/female), duration of chronic hepatitis B, and duration of adefovir treatment in patients with or without osteomalacia (*p* values were 0.070, 0.367, 0.791, respectively), and patients with osteomalacia were older than patients without (*p* = 0.001). The median duration of ADV treatment before the onset of osteomalacia was 6.5 years, while the minimum duration was only 1.5 years, and the maximum was 17 years. The clinical symptoms of patients with osteomalacia included muscle weakness/fatigue (100%), bone pain (100%) (primarily in the heel, then spreading to the lower back and ribs), and difficulty walking [66.4% (93 of 140 patients)]. Because of the nonspecific symptoms, 33.6% (47 of 140 patients) patients were misdiagnosed. Of these patients, 31 were misdiagnosed with osteoporosis, five were misdiagnosed with spondyloarthropathy, and one was misdiagnosed with multiple myeloma.

**TABLE 2:** Characteristics of the study population.

Characteristics	Patients with osteomalacia (*n* = 140)	Patients without osteomalacia (*n* = 53)	*p*	Normal range
Age, years	58.0 (47.0–63.8)	49.5 (39.0–55.0)	0.001	
Gender, male/female	83/57	30/10	0.070	
Duration of chronic hepatitis B, years	12.0 (8.0–20.0)	10.0 (8.0–20.0)	0.367	
Duration of adefovir treatment, years	6.5 (5.0–8.0)	6.0 (5.0–8.5)	0.791	
Blood gas analysis				
Metabolic acidosis	46 (39.3)	1 (5.2)	<0.001	
Blood parameters				
ALT (U/L)	18.0 (12.0–26.3)	28.0 (15.8–41.3)	<0.001	0–65
AST (U/L)	22.0 (17.0–27.5)	26.0 (21.0–30.5)	0.006	8–37
ALP (U/L)	245.0 (182.5–316.0)	86.0 (66.0–105.0)	<0.001	15–112
Cr (μmol/L)	95.0 (82.0–110.0)	68.5 (58.5–76.8)	<0.001	53–115
e-GFR [mL/(min*1.73m^2^)]	65.0 (54.0–75.0)	106.1 (92.1–112.9)	<0.001	≥90
UA (μmol/L)	120.0 (94.5–141.5)	305.5 (246.8–358.3)	<0.001	210–430
Ca (mmol/L)	2.30 (2.22–2.38)	2.43 (2.32–2.38)	<0.001	2.08–2.60
P (mmol/L)	0.54 (0.45–0.64)	0.97 (0.91–1.08)	<0.001	0.80–1.60
Hypokalemia (mmol/L)	22 (28.2)	2 (4.4)	0.025	
Urine parameters				
Urine pH	6.5 (6.0–7.0)	6.0 (6.0–7.0)	0.436	4.6–8.0
Nondiabetic glucosuria	88 (71.5)	0 (0.0)	<0.001	
Proteinuria	105 (84.7)	3 (6.3)	<0.001	

The data are presented as median (interquartile range) and n (%).

ALP: alkaline phosphatase; ALT: alanine aminotransferase; AST: aspartate aminotransferase; Ca: calcium; Cr: creatinine; e-GFR: estimated glomerular filtration rate; P: phosphorus; UA: uric acid.

### Laboratory Tests and Imaging Examination

The laboratory results of the patients were presented in Table 2. The laboratory results of the case group showed that 94.9% of patients had hypophosphatemia [0.54 (0.45–0.64) mmol/L], 92.7% of patients had high ALP levels [245 (182.5–316) U/L], and 90.6% of patients had hypouricemia [122 (94.5–141.5) μmol/L]. Nondiabetic glycosuria occurred in 71.5% of the patients, proteinuria occurred in 84.7% of the patients. In addition, serum OC in 62.9% of patients and serum β-CTX in 77.3% of patients increased, suggesting that bone metabolism was active. 17.6% of patients had elevated serum creatinine, indicating that these patients developed renal insufficiency. 52.7% of patients had a slight decrease in estimated glomerular filtration rate (eGFR), and 37.9% of patients had eGFR less than 60. For these patients, it is necessary to pay close attention to their renal function. The patients without osteomalacia had no abnormalities in laboratory tests. The bone turnover markers were evaluated based on the reference range we previously established (Hu et al., 2013).

Radiography tests were carried out on 116 patients of the case group. Fractures were verified in 68 of 116 (58.6%) patients. These fractures consisted of vertebral compression fractures (39 of 116 patients), rib fractures (26 of 116 patients), pelvic deformities (16 of 116 patients) and pseudo-fractures of pubis, femur, ulna and tibia (10 of 116 patients). It is worth mentioning that 23 of 116 patients suffered from multiple fractures. 39 patients with osteomalacia underwent a whole-body bone scan by SPECT/CT, 38 of whom demonstrated high uptake in multiple locations throughout the skeleton. The results of the BMD examination revealed 86.4% patients were osteoporosis and 13.6% patients were osteopenia in the case group.

### Treatment and Follow-Up

ADV administration ceased immediately after diagnosis, and entecavir was administered as the hepatitis B antiviral therapy for some of the patients. Calcium carbonate (1.5 g/d) and calcitriol (0.5 μg/d) were also administered. Sodium bicarbonate (1.5 g/d) was used for patients with compensated or decompensated metabolic acidosis. Notably, elemental phosphate supplementation was not used for patients.

**FIGURE 1:**
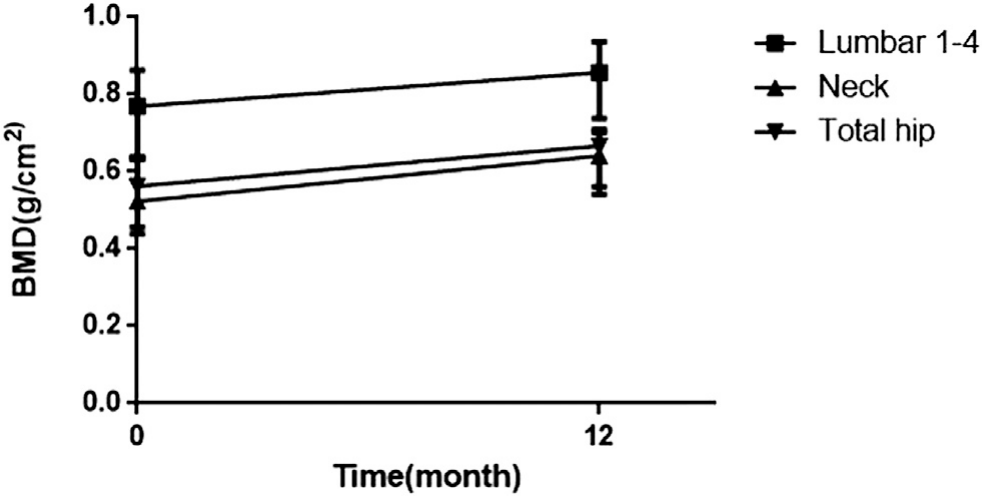
Changes in bone mineral density (baseline and post-treatment). BMD = bone mineral density.

Patients in the control group did not participate in follow-up. The average follow-up period was 11.7 months (1–111 months). During the follow-up, symptoms (including bone pain and activity function) were relieved within 3 to 6 months. Hypophosphatemia, nondiabetic glucosuria and proteinuria recovered rapidly, and 63, 76.3, and 50% of patients had returned to normal by 6 months, respectively. The recovery of serum ALP and blood uric acid was slow. At 18 months, 21.4 and 18.6% of patients returned to normal, respectively. The recovery of renal function in patients is relatively difficult. Serum creatinine returned to normal in 39.1% of patients at the 12-months re-examination, and eGFR in 20.8% of patients returned to normal. After treatment, the serum ALP and β-CTX concentration gradually decreased, and the OC concentration gradually increased, indicating that the patient’s bone turnover state became stable, and the bone turnover markers returned to normal relatively slowly. The changes of biochemical parameters are shown in Table 3. 19 patients underwent X-ray radiographs after treatment. None of these patients had new fractures. X-rays of five of them indicated that the fracture lines were healing. BMD was measured on 24 patients, and the results showed that 100% of patients had improved BMD compared to before treatment. As shown in Figure 1, the BMD of the lumbar 1-4, total hip, and femoral neck was significantly improved.

**TABLE 3:** Changes in biochemical parameters (baseline and post-treatment) of the patients with osteomalacia.

Characteristics of the case group	Baseline (*n* = 140)	3 months (*n* = 73)	6 months (*n* = 47)	12 months (*n* = 35)	18 months (*n* = 14)	Normal range
P (mmol/L)	0.54 (0.45–0.64)	0.77 (0.68–0.90)	0.89 (0.74–1.04)	0.84 (0.74–1.04)	0.94 (0.66–1.05)	0.80–1.60
ALP (U/L)	246 (191–316)	277 (204.5–351.5)	242 (167.5–357)	179 (130–215)	140 (115–208)	15–112
UA (μmol/L)	122 (97–143)	164 (135–186)	171 (138–203)	177 (152–209)	170 (147–208)	210–430
OC (ng/ml)	28.28 (23.21–37.57)	55.3 (45.3–84.1)	62.3 (44.4–93.1)	52.2 (40.4–65.1)	45.2 (32.7–54.7)	Female:4.91–22.31
						Male:5.58–28.62
β-CTX (ng/L)	1031 (603.6–1476.3)	2017 (1317–2836)	1904 (1294–2785)	1178 (916–1648)	1050 (895–1550)	Female:112–497
						Male:100–612
Nondiabetic glucosuria	88 (71.5)	20 (34.5)	9 (23.7)	5 (19.2)	2 (16.7)	
Proteinuria	105 (84.7)	43 (74.1)	19 (50.0)	12 (44.4)	6 (50.0)	

The data are presented as median (interquartile range) and n (%). n (%) are the abnormal cases (abnormal cases/available cases).

ALP: alkaline phosphatase; β-CTX: beta C-terminal cross-linked telopeptides of type l collagen; OC: osteocalcin; P: phosphorus; UA: uric acid.

### Association Between SNVs in Drug Transporter Genes and the Risk of ADV-Induced Osteomalacia

We compared the genotypes and alleles of the case group with the control group. The SNVs that differed in genotype and allele distribution between groups were listed in Supplementary Table S1. 13 SNVs of five genes (*ABCB1, ABCG2, SLC28A3, SLC34A1,* and *SLC O 4A1*) were distributed differently between the two groups. Among these sites, 5 SNVs were located in the intron region, 5 SNVs were located in the 3′UTR region, and the remaining 3 sites were located in the exon region. The rs2231142 (c.421G > T, p. Q141K) is a nonsynonymous variant of *ABCG2* gene, rs1047099 (c.232G > A, p. V78I) and rs3195701 (c.549G > T, p. G183G) are nonsynonymous and synonymous variant of *SLC O 4A1* gene. Regrettably, after adjusting with the Benjamini-Hochberg method, none of these SNVs had a different distribution.

Regression analysis was used to determine the predictors of osteomalacia risk in the study population. As mentioned earlier, there was no significant difference in sex ratio, duration of chronic hepatitis B, and duration of adefovir treatment in patients with or without osteomalacia. When compared with the control group, old age [per 1 year, odds ratio (OR), 1.053; 95% confidence interval (95% CI), 1.020–1.087; *p* = 0.015] was associated with osteomalacia. Through high-throughput sequencing, we found 39 SNVs associated with osteomalacia risk (Supplementary Table S2). These 39 sites were concentrated in 10 genes (*ABCB1, ABCB11, ABCG2, SLC4A8, SLC9A3, SLC14A2, SLC15A2, SLC28A3, SLC34A1,* and *SLC O 4A1*). Among these sites, 15 SNVs were located in the intron region, 10 SNVs were located in the 3′UTR region, two sites were splicing site, and the remaining 3 sites were located in the exon region. Unfortunately, the FDR-adjusted *p* values of these sites were not statistically significant.

## Discussion

ADV is a commonly used drug for the treatment of chronic hepatitis B. However, prospective studies have shown that dose-dependent nephrotoxicity is associated with ADV, while ADV 10 mg/d is relatively safe for the treatment of chronic hepatitis B (Izzedine et al., 2004). In recent years, a growing number of studies have reported that KTD could be associated with low-dose (10 mg/d), long-term application of ADV (Wong et al., 2010; Kim et al., 2012; Law et al., 2013; Wang et al., 2015; Xu et al., 2015), which can lead to osteomalacia.

Here, we present the clinical features of 140 patients with ADV-associated osteomalacia and 53 ADV-treated chronic hepatitis B patients who did not suffer from osteomalacia. ADV-induced osteomalacia may occur at the therapeutic dose after long-term application, mainly after >5 years. The median duration of ADV treatment before the onset of osteomalacia of our patients was 6.5 years. The clinical symptoms of ADV-induced osteomalacia frequently manifest as weakness and bone pain. Pain often begins in the lower limbs then gradually spreads to the entire body. The biochemical manifestations are hypophosphatemia, high ALP, hypouricemia, nondiabetic glycosuria, proteinuria, metabolic acidosis and high bone turnover markers. The main treatments for ADV-induced osteomalacia are withdrawing ADV and administering calcitriol and calcium. Notably, elemental phosphate supplementation is not necessary. We followed these patients for an average of 11.7 months (1–111 months). During the follow-up, the patients experienced significant improvements in clinical symptoms and biochemical indicators. Symptoms were relieved within 3 to 6 months. At 6 months, serum phosphorus returned to normal in 63% of patients, nondiabetic diabetes and proteinuria turned negative in 76.3 and 50% of patients, respectively. The recovery of serum ALP and blood uric acid was not as rapid as the indicators mentioned before. At 18 months, 21.4 and 18.6% of patients returned to normal, respectively. At 12 months, serum creatinine returned to normal in 39.1% of patients, and eGFR returned to normal in 20.8% of patients. In general, the prognosis of ADV-induced osteomalacia is good. After standard treatment, the patients’ clinical symptoms can disappear completely and their biochemical indicators will gradually recover.

Our study found that osteomalacia occurs in 72.5% of patients after long-term use of ADV. Patients who did not suffer from this adverse reaction were not susceptible to ADV nephrotoxicity. This finding indicated that genetic factors may play an important role in the metabolism of ADV. Due to the potentially severe nephrotoxicity of ADV and the known susceptibility of certain people, it is important to identify possible risk factors. In this study, we report the results of a study of pharmacogenetic determinants of ADV-associated osteomalacia in patients with chronic hepatitis B. There was no significant difference in sex ratio (male/female), duration of chronic hepatitis B, and duration of adefovir treatment in patients with or without osteomalacia (*p* values were 0.070, 0.367, 0.791, respectively). Therefore, through regression analysis, we found that age was a risk factor for osteomalacia (per 1 year, OR, 1.053; 95% CI, 1.020–1.087; *p* = 0.015), which is consistent with previous studies (Rodriguez-Novoa et al., 2009; Salvaggio et al., 2017). We also found 13 SNVs distributed differently between the patients with and without osteomalacia. Through regression analysis, 39 SNV sites were found associated with the risk of osteomalacia in ADV-treated hepatitis B patients. But after we used the Benjamini-Hochberg method to correct the *p* values, the relationship between these sites and the occurrence of the disease disappeared. In other words, this study failed to find genetic predictors of osteomalacia in HBV-infected patients treated with ADV.

So far, the pharmacogenetic research of ADV has mainly involved the efficacy of anti-hepatitis B virus. Studies of the relationship between different genotypes and adverse reactions mainly focus on its similar drug tenofovir. Rodríguez-Nóvoa (Rodriguez-Novoa et al., 2009) reported 19 cases of KTD in 115 Spanish human immunodeficiency virus (HIV)-infected patients treated with tenofovir. They analyzed the relationship between single nucleotide polymorphisms (SNPs) of five transporter genes and this adverse reaction. It was found that those who carrying C allele at rs717620 locus of the *ABCC2* gene had a high risk of developing KTD. Nishijima et al. (Nishijima et al., 2012) treated 190 HIV- infected patients with tenofovir, 19 of whom had KTD. They then tested 14 SNPs of 5 transporter genes, and found C allele at rs717620 locus and A allele at rs2273697 locus of the *ABCC2* gene could predicted higher risk of KTD. Our department has analyzed 76 ADV-induced osteomalacia patients (Wei et al., 2016), and revealed a higher percentage of the GA genotype at rs3740070 of the *ABCC2* gene in osteomalacia patients than in healthy people. In the previous study, we did not include patients who had taken ADV but did not develop osteomalacia as the control group, so the results of the two studies may be different. As for other studies on tenofovir were conducted in HIV-infected populations, they are different from the research populations and genetic backgrounds of this study. In addition, the purpose of this study is to discover the genetic risk factors for osteomalacia, while the target population of the previous studies were patients with renal tubular injury. The above studies have selected transporter encoding genes closely related to tenofovir or ADV uptake and excretion, including *ABCC2, ABCC4, ABCC10, SLC22A6* and *ABCB1*. Although we have also selected these genes, the relationship between the genetic variation of some of the transporters selected in our study and ADV adverse reactions has not been reported. These genes may cause some interference in the analysis of the results. none of the previous studies performed FDR correction on the *p* value. In multiple comparisons, adjusted-*p* value can assess the relationship between gene variants and disease risk more accurately. The occurrence of disease is the result of the combined effect of genetic factors and environmental factors. This study failed to find that the genetic risk factors of ADV-induced osteomalacia may be due to heredity having less influence on the occurrence of the disease. This study may not include enough subjects to discover genetic risk factors. The sample size of this study is already the largest in existing studies, and no positive results can be found. In the future, the sample size needs to be further expanded in order to study the risk factors of this disease.

To our knowledge, there is no previous report of ADV-induced osteomalacia with such a large sample size, not to mention so many controls. This study is the largest study of ADV-induced osteomalacia so far. Most of the previous studies only examined a limited number of polymorphisms in some candidate transporters. In this study, the target capture sequencing method was used to detect variations in 51 drug transporter genes, taking into account some of the previously unreported transporters. However, there are several limitations of our study. First, we lacked the baseline data of patients before the initiation of ADV. Second, the control group accounted for 27.5% of our patients, and we did not follow up the patients of control group. In further study we can expand the control group and follow up the control group to see if they will develop osteomalacia during the follow-up.

In conclusion, long-term (>5 years) low-dose (10 mg/d) ADV treatment is one of the most important causes of adult-onset osteomalacia in China. Clinicians should be aware of this side effect when using ADV for therapy. We recommend routine monitoring of serum ALP and serum phosphorus levels during ADV treatment. Urine routine and arterial blood gas tests are also needed when necessary. We failed to find genetic variants that can predict the risk of osteomalacia in present study. Nevertheless, further study of individual differences in ADV tolerance is necessary.

## Data Availability

The datasets presented in this study can be found in online repositories. The names of the repository/repositories and accession number(s) can be found below: https://www.ncbi.nlm.nih.gov/sra/PRJNA713961.
