# Supplementary material for: Follow-Up of Adefovir Dipivoxil Induced Osteomalacia: Clinical Characteristics and Genetic Predictors
Source: Front Pharmacol. 2021 Apr 28;12:636352. doi: 10.3389/fphar.2021.636352 (PMC8113870; doi:10.3389/fphar.2021.636352)
Supplement: Supplementary file 1 [file DataSheet1.DOCX]

**Supplementary Table 1.** Genotypes and allelic frequencies at drug transporter genes in the patients with and without osteomalacia

| Gene (protein), position, SNP identification, and genotype or allele | Patients with osteomalacia (n=106) | Patients without osteomalacia (n=52) | *p* | *p^a^* |
| --- | --- | --- | --- | --- |
| *ABCB1* |  |  |  |  |
| g.87230435T>C, rs2188524 | | | |  |
| T/T | 94 | 38 | **0.013** |  |
| T/C | 12 | 14 |  |  |
| C/C | 0 | 0 |  |  |
| T | 200 | 90 | **0.018** | 0.507 |
| C | 12 | 14 |  |  |
| *ABCG2* |  |  |  |  |
| g.89015857C>T, rs2231164 | | | |  |
| C/C | 27 | 10 | **0.030** |  |
| C/T | 62 | 24 |  |  |
| T/T | 17 | 18 |  |  |
| C | 116 | 44 | **0.008** | 0.596 |
| T | 96 | 60 |  |  |
| g.89052323G>T (c.421G>T), rs2231142 | | | |  |
| G/G | 26 | 24 | **0.022** |  |
| G/T | 58 | 21 |  |  |
| T/T | 22 | 7 |  |  |
| G | 110 | 69 | **0.015** | 0.507 |
| T | 102 | 35 |  |  |
| *SLC28A3* |  |  |  |  |
| g.86890931T>A, rs11140489 | | | |  |
| T/T | 72 | 45 | **0.032** |  |
| T/A | 30 | 7 |  |  |
| A/A | 4 | 0 |  |  |
| T | 174 | 97 | **0.007** | 0.507 |
| A | 38 | 7 |  |  |
| g.86892287G>A, rs4877272 | | | |  |
| G/G | 72 | 45 | **0.032** |  |
| G/A | 30 | 7 |  |  |
| A/A | 4 | 0 |  |  |
| G | 174 | 97 | **0.007** | 0.507 |
| A | 38 | 7 |  |  |
| g.86892894A>C, rs3750406 | | | |  |
| A/A | 72 | 45 | **0.032** |  |
| A/C | 30 | 7 |  |  |
| C/C | 4 | 0 |  |  |
| A | 174 | 97 | **0.007** | 0.507 |
| C | 38 | 7 |  |  |

**Supplementary Table 1** (continued)

| Gene (protein), position, SNP identification, and genotype or allele | Patients with osteomalacia (n=106) | Patients without osteomalacia (n=52) | *p* | *p^a^* |
| --- | --- | --- | --- | --- |
| g.86893071T>C, rs7858075 | | | |  |
| T/T | 72 | 45 | **0.032** |  |
| T/C | 30 | 7 |  |  |
| C/C | 4 | 0 |  |  |
| T | 174 | 97 | **0.007** | 0.507 |
| C | 38 | 7 |  |  |
| g.86895853T>C, rs10868135 | | | |  |
| T/T | 71 | 44 | **0.042** |  |
| T/C | 30 | 8 |  |  |
| C/C | 5 | 0 |  |  |
| T | 172 | 96 | **0.009** | 0.507 |
| C | 40 | 8 |  |  |
| g.86909082A>G, rs10868137 | | | |  |
| A/A | 71 | 44 | **0.042** |  |
| A/G | 30 | 8 |  |  |
| G/G | 5 | 0 |  |  |
| A | 172 | 96 | **0.009** | 0.507 |
| G | 40 | 8 |  |  |
| *SLC34A1* |  |  |  |  |
| g.176817143G>T, rs3812035 | | | |  |
| G/G | 50 | 31 | **0.045** |  |
| G/T | 41 | 20 |  |  |
| T/T | 15 | 1 |  |  |
| G | 141 | 82 | **0.024** | 0.507 |
| T | 71 | 22 |  |  |
| g.176817583A>T, rs55785724 | | | |  |
| A/A | 50 | 31 | **0.045** |  |
| A/T | 41 | 20 |  |  |
| T/T | 15 | 1 |  |  |
| A | 141 | 82 | **0.024** | 0.507 |
| T | 71 | 22 |  |  |
| *SLCO4A1* |  |  |  |  |
| g.61288038G>A (c.232G>A), rs1047099 | | | |  |
| G/G | 75 | 24 | **0.007** |  |
| G/A | 25 | 25 |  |  |
| A/A | 6 | 3 |  |  |
| G | 175 | 73 | **0.012** | 0.507 |
| A | 37 | 31 |  |  |
| g.61288355G>T (c.549G>T), rs3195701 | | | |  |
| G/G | 72 | 24 | **0.022** |  |

**Supplementary Table 1** (continued)

| Gene (protein), position, SNP identification, and genotype or allele | Patients with osteomalacia (n=106） | Patients without osteomalacia (n=52) | *p* | *p^a^* |
| --- | --- | --- | --- | --- |
| G/T | 28 | 25 |  |  |
| T/T | 6 | 3 |  |  |
| G | 172 | 73 | **0.029** | 0.556 |
| T | 40 | 31 |  |  |

The data are no. of patients for genotypes and no. of alleles for alleles. Statistically significant pvalues (p<0.05) are shown in boldface.

^a^*p* adjusted by Benjamini-Hochberg method.

**Supplementary Table 2.** Predictors of the risk of osteomalacia in hepatitis B virus infected patients treated with adefovir dipivoxil

| Characteristic | OR | 95%CI | *p* | *p^a^* |
| --- | --- | --- | --- | --- |
| Female sex | 1.423 | 0.501-4.040 | 0.508 | NA |
| Age (per year) | 1.053 | 1.020-1.087 | **0.015** | NA |
| Duration of chronic hepatitis B (years) | 0.998 | 0.953-1.044 | 0.927 | NA |
| Duration of adefovir treatment (years) | 0.997 | 0.852-1.167 | 0.972 | NA |
| Genetic polymorphisms^b^ |  |  |  |  |
| *ABCB1* |  |  |  |  |
| rs2188524 C allele | 0.347 | 0.147-0.817 | **0.016** | 0.596 |
| *ABCB11* |  |  |  |  |
| rs496550 C allele | 1.656 | 1.001-2.738 | **0.049** | 0.669 |
| rs495714 T ellele | 1.674 | 1.016-2.757 | **0.043** | 0.655 |
| *ABCG2* |  |  |  |  |
| rs2231164 T allele | 0.572 | 0.344-0.953 | **0.032** | 0.620 |
| rs2231142 T allele | 1.871 | 1.127-3.106 | **0.015** | 0.596 |
| *SLC4A8* |  |  |  |  |
| rs34555885 T allele | 2.722 | 1.097-6.755 | **0.031** | 0.620 |
| rs57024323 T allele | 2.536 | 1.045-6.153 | **0.040** | 0.655 |
| rs2292220 T allele | 0.524 | 0.294-0.937 | **0.029** | 0.620 |
| rs17125880 T allele | 2.722 | 1.097-6.755 | **0.031** | 0.620 |
| *SLC9A3* |  |  |  |  |
| rs3777233 T allele | 0.420 | 0.177-0.998 | **0.050** | 0.669 |
| *SLC14A2* |  |  |  |  |
| rs1484873 G allele | 2.454 | 1.133-5.311 | **0.023** | 0.609 |
| *SLC15A2* |  |  |  |  |
| rs3215370 T allele | 2.018 | 1.085-3.752 | **0.027** | 0.620 |
| rs9812515 A allele | 2.018 | 1.085-3.752 | **0.027** | 0.620 |
| rs2293616 A allele | 2.080 | 1.116-3.876 | **0.021** | 0.596 |
| rs2293615 G allele | 2.080 | 1.116-3.876 | **0.021** | 0.596 |
| rs3817601 T allele | 2.080 | 1.116-3.876 | **0.021** | 0.596 |
| rs3215371 T allele | 1.941 | 1.053-3.578 | **0.034** | 0.624 |
| rs2293611 C allele | 2.608 | 1.036-6.566 | **0.042** | 0.655 |
| rs2257109 T allele | 2.080 | 1.116-3.876 | **0.021** | 0.596 |
| rs2257115 A allele | 2.080 | 1.116-3.876 | **0.021** | 0.596 |
| rs2257212 T allele | 2.080 | 1.116-3.876 | **0.021** | 0.596 |
| rs2257214 C allele | 2.080 | 1.116-3.876 | **0.021** | 0.596 |
| rs1143670 G allele | 2.080 | 1.116-3.876 | **0.021** | 0.596 |
| rs3762819 A allele | 2.080 | 1.116-3.876 | **0.021** | 0.596 |
| rs1143671 T allele | 2.080 | 1.116-3.876 | **0.021** | 0.596 |
| rs1143672 A allele | 2.080 | 1.116-3.876 | **0.021** | 0.596 |
| rs1920310 T allele | 4.631 | 1.049-20.44 | **0.043** | 0.655 |
| *SLC28A3* |  |  |  |  |
| rs11140489 A allele | 2.978 | 1.272-6.973 | **0.012** | 0.596 |

**Supplementary Table 2** (continued)

| Characteristic | OR | 95%CI | *p* | *p^a^* |
| --- | --- | --- | --- | --- |
| rs4877272 A allele | 2.978 | 1.272-6.973 | **0.012** | 0.596 |
| rs3750406 C allele | 2.978 | 1.272-6.973 | **0.012** | 0.596 |
| rs7858075 C allele | 2.978 | 1.272-6.973 | **0.012** | 0.596 |
| rs10868135 C allele | 2.690 | 1.210-5.981 | **0.015** | 0.596 |
| rs7853758 A allele | 2.391 | 1.055-5.418 | **0.037** | 0.646 |
| rs10868137 G allele | 2.690 | 1.055-5.418 | **0.015** | 0.596 |
| *SLC34A1* |  |  |  |  |
| rs5030873 C allele | 1.785 | 1.025-3.108 | **0.041** | 0.655 |
| rs3812035 T allele | 1.819 | 1.055-3.134 | **0.031** | 0.620 |
| rs55785724 T allele | 1.819 | 1.055-3.134 | **0.031** | 0.620 |
| *SLCO4A1* |  |  |  |  |
| rs1047099 A allele | 0.513 | 0.297-0.888 | **0.017** | 0.596 |
| rs3195701 T allele | 0.555 | 0.322-0.958 | **0.034** | 0.624 |

Statistically significant pvalues (*p*<0.05) are shown in boldface.

NA: not available; OR: odds ratio; 95%CI: 95% confidence interval.

^a^*p* adjusted by Benjamini-Hochberg method.

Only the statistically significant alleles are listed here.
